# Metagenomics next-generation sequencing assists in the diagnosis of infant pertussis encephalopathy: A case report

**DOI:** 10.1097/MD.0000000000033080

**Published:** 2023-02-22

**Authors:** Haiyang Zhang, Xiao Wang, Han Xia, Zhongqiang Liu

**Affiliations:** a Department of Pediatric Intensive Care Unit, West China Second University Hospital, Sichuan University; Key Laboratory of Birth Defects and Related Diseases of Women and Children (Sichuan University), Ministry of Education, Chengdu, China; b Department of Scientific Affairs, Hugobiotech Co., Ltd., Beijing, China.

**Keywords:** *Bordetella pertussis*, diagnosis, encephalopathy, infant, mNGS

## Abstract

**Rationale:**

Pertussis is an acute respiratory infection that often occurs in the pediatric population, especially in infants under 3 months old. *Bordetella pertussis* is the causative agent of pertussis, which can lead to pneumonia, encephalopathy, and pulmonary hypertension, causing death in severe cases. Therefore, an accurate and comprehensive diagnosis of the pathogen is essential for effective treatment.

**Patient concerns:**

We report a case of 2-month-old male infant admitted to the pediatric intensive care unit of West China Second University due to hoarse cough for 7 days, accompanied by a crowing-like echo, fever and listlessness, occasional nonprojectile vomiting with anorexia, shortness of breath, accelerated heart rate, cyanosis of the lips, and convulsions. *B pertussis* was identified by metagenomic next-generation sequencing in blood and cerebrospinal fluid and polymerase chain reaction assay using blood.

**Diagnoses:**

The infant was diagnosed with pertussis.

**Interventions:**

Intravenous infusion of erythromycin (50 mg/kg/d) for anti-infection and dexamethasone for alleviating intracranial inflammatory reaction were given.

**Outcomes:**

The patient was eventually recovered and discharged.

**Lessons:**

This case report emphasized the importance of metagenomic next-generation sequencing using cerebrospinal fluid and blood for early diagnosis of pertussis-associated encephalopathy.

## 1. Introduction

Pertussis is an acute and highly contagious respiratory disease caused by *Bordetella pertussis* or *Bordetella parapertussis*, which was the leading cause of morbidity and mortality in children in the first half of the 20th century.^[1]^ Although the incidence has decreased significantly after the introduction of pertussis vaccine, it has not been completely eliminated and may be on the rise again in some areas. In 2014, the WHO reported 139,786 cases; in the United States, a total of 61,430 pertussis cases were reported during 2013 to 2014. Although the vast majority of these patients were immunized.^[2]^ In particular, infants under 3 months old, who are too young to be immunized, have the highest morbidity and mortality rates, and affected infants require treatment in pediatric intensive care unit.^[3,4]^ In addition to causing respiratory diseases, *B pertussis* may also cause pertussis encephalopathy. This condition is usually seen in children under 2 years old, and if not treated promptly and effectively, it may cause cerebral hypoxia, leading to convulsions and other symptoms in young children.^[5]^

In this case, we reported a rare case of pertussis encephalopathy in a 2-month-old infant. *B pertussis* was identified by metagenomic next-generation sequencing (mNGS) in blood and cerebrospinal fluid (CSF), which was confirmed by polymerase chain reaction (PCR) using blood.

## 2. Case description

A 2 months and 12 days old male infant was admitted to the pediatric intensive care unit of West China Second University on March 18th, 2022. On admission, the infant presented mainly with hoarse cough for 7 days accompanied by a crowing-like echo, fever and listlessness, occasional nonprojectile vomiting with anorexia, shortness of breath, accelerated heart rate, cyanosis of the lips and convulsions. Due to the acute onset and rapid progression of the disease, the patient was given oxygen inhalation, piperacillin tazobactam intravenous infusion (100 mg/kg/d), and nebulization in our emergency department, but the effect was unsatisfactory. Afterwards, he developed recurrent convulsions and required ventilator-assisted ventilation, and after 2 resuscitations, his condition began to improve. This is a baby conceived by in vitro fertilization with a normal birth history. He was discharged from our neonatal intensive care unit on the 12th day of life after improvement following phototherapy for neonatal hyperbilirubinemia. This child had not received any vaccination of pertussis since birth. In the family history, his mother had been vaccinated against whooping cough as a child, but she had not been vaccinated again during pregnancy.

**Figure 1.**
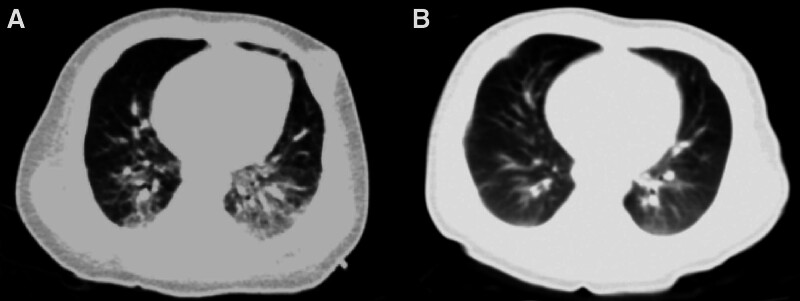
Chest CT results of the patient. A: Chest CT (on admission): Inflammatory alterations in the lung with interstitial changes and scattered emphysema sign; B: Reexamination of chest CT (20 days after admission): A little inflammation in both lungs, significantly reduced pulmonary interstitial changes compared with before. CT = computed tomography.

**Figure 2.**
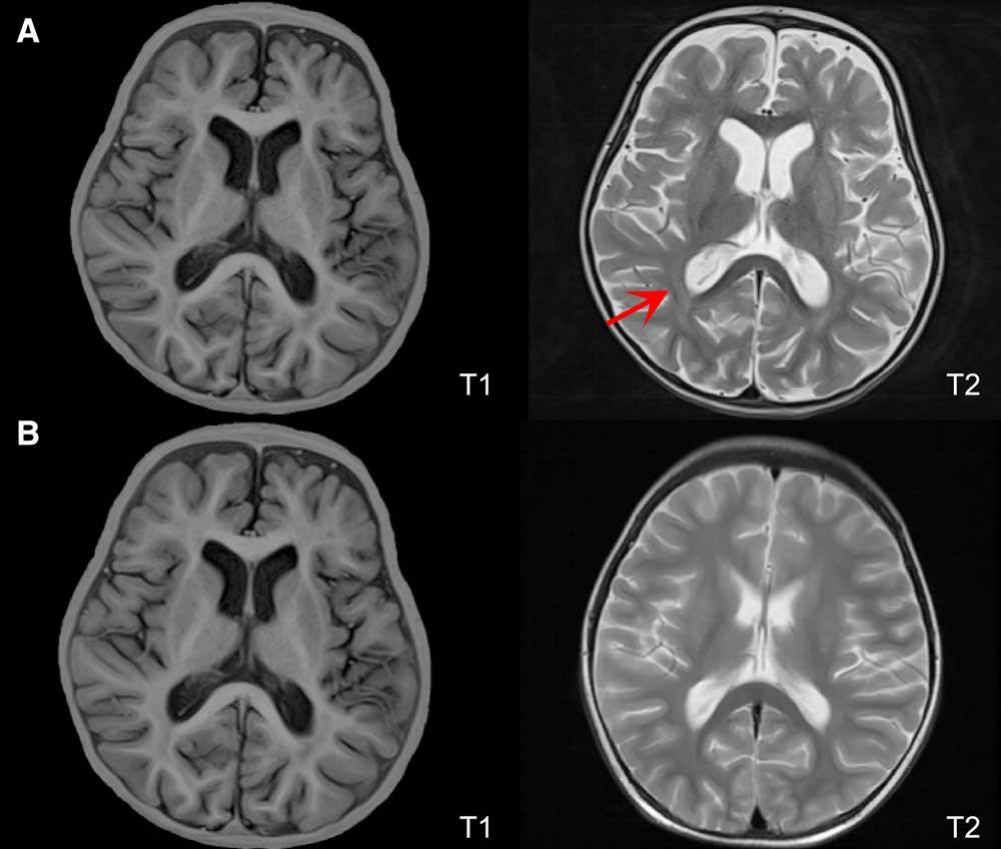
Cranial MRI result of the patient. A: Cranial MRI (on admission): Slightly wide bilateral lateral ventricles, slightly wide and deepened sulcal fissures, slightly wide bilateral temporal pole extracellular space, abnormal signals in the white matter area next to the lateral ventricles; B: Reexamination of cranial MRI (20 d after admission): Slightly wide sulcus in part of bilateral cerebral hemispheres and disappearance of abnormal signal in the paraventricular white matter area. MRI = magnetic resonance imaging.

Physical examination at admission: temperature 37.3°C, respiratory rate 56 times/min, pulse rate 172 times/min, blood pressure 96/50 mm Hg. His Glasgow Coma score was 13 (E4M6V3). The fontanel was not closed (2 × 2 cm) with fontanel bulge and slightly elevated tension. Three-concave sign was positive, bilateral breath sounds were rough. Laboratory blood examination showed the following results: white blood cell count (WBC) 6.5 × 10^9^/L, percentage of lymphocytes 55.2%, neutrophilic granulocyte percentage 33.3%, blood platelet 280 × 10^9^/L, hemoglobin 84 g/L, C reactive protein 15.7 mg/L, procalcitonin 13.28 ng/mL. CSF examination showed a nucleated cell count of 34 × 10^6^/L (neutrophil granulocytes 61%, lymphocytes 30.0%, monocytes 9.0%). Total protein of CSF is 1190.0 mg/L and CSF glucose is 5.30 mmol/L. Blood gas analysis, liver and kidney function and electrolytes were unremarkable. The echocardiogram was completely normal. Chest computed tomography showed inflammatory alterations in the lung with interstitial changes and scattered emphysema sign (Fig. 1A). Cranial magnetic resonance imaging revealed slightly wide bilateral lateral ventricles, slightly wide and deepened sulcal fissures, slightly wide bilateral temporal pole extracellular space, and abnormal signals in the white matter area next to the lateral ventricles (Fig. 2A).

**Figure 3.**
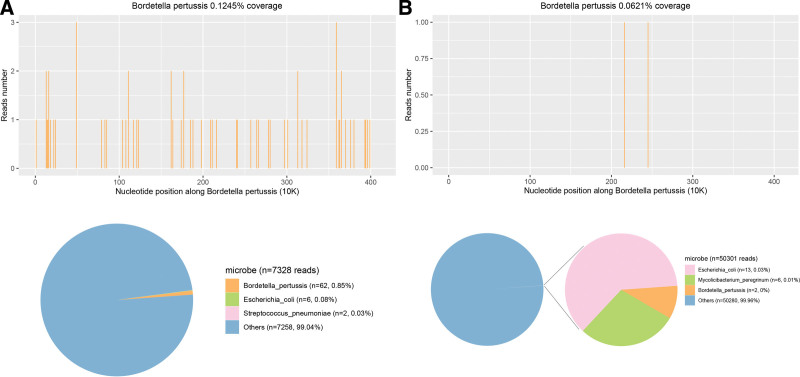
The coverage and abundance of *Bordetella pertussis* detected by mNGS using blood (A) and cerebrospinal fluid (B) on the 3rd day of admission. mNGS = metagenomic next-generation sequencing.

According to the above test parameters and clinical symptoms, the patient was preliminarily diagnosed with severe pneumonia, intracranial infection, and sepsis, accompanied by respiratory failure, anemia, metabolic acidosis, and hyperlactatemia. The patient was timely empirically given meropenem combined with vancomycin for antiinfection, gamma globulin therapy and other symptomatic treatments. On the 2nd day of admission, *B pertussis* PCR of serum was positive and sputum respiratory multiplex nucleic acid test (PCR assay, including Human rhinovirus, Human orthopneumovirus, Adenovirus, Parainfluenza virus, Human metapneumovirus, Influenza A/B virus, Coronavirus, Bocavirus, *Mycoplasma pneumoniae, Chlamydia*) was negative; serum G ((1-3)-β-d-glucan) tests and galactomannan test were negative. On the 3rd day of admission, reexamination of laboratory blood report showed that C reactive protein increased to 110.1 mg/L and procalcitonin was 7.48 ng/mL. The coagulation function was still abnormal, and the effect of antiinfection therapy was poor. At the same time, PACEseq mNGS (Hugobiotech, Beijing, China) of blood and CSF detected *B pertussis* with 62 and 2 specific sequences, respectively (Fig. 3). Accordingly, the infant patient was referred with a diagnosis of pertussis encephalopathy with severe pertussis pneumonia caused by *B pertussis*.

We confirmed this diagnosis in a PCR verification of the blood sample left by the patient after discharge from the hospital. The DNA was extracted using a microsample genomic DNA extraction kit (Qiagen, Germany) and the DNA product was detected after PCR amplification (95°C, 10 minutes; 95°C, 30 seconds, 51°C, 30 seconds, 72°C, 30 seconds, 40 cycles; 72°C, 1 minute). Through sanger sequencing, the DNA product was identified as *B pertussis* with 100% sequence identity.

After diagnosis, the medication was promptly adjusted by replacing meropenem and vancomycin with intravenous infusion of erythromycin (50 mg/kg/d) for antiinfection and adding dexamethasone to alleviate intracranial inflammatory reaction. At this time, *B pertussis* was detected in the CSF, blood flow of the child, so it was necessary to be alert to the possibility of immune deficiency in the child at that time, and it was easy to combine invasive fungal disease. Micafungin (3 mg/kg/d) was used to prophylactic empirical antifungal therapy.

On the fifth day of admission, reexamination of laboratory blood tests showed that inflammatory parameters decreased than before, and the coagulation disorders improved; generally normal assisted invasive ventilation, and the current treatment was continued. Fourteen days after admission, the infant patient’s condition improved, with stable vital signs, normalized blood count and inflammatory parameters, but still required ventilator-assisted ventilation. On the 20th day of admission, a retest for *B pertussis* nucleic acid in the blood was negative. Considering that the current antipertussis treatment was effective and sequential treatment was required, erythromycin and micafungin was discontinued and switched to oral azithromycin (10 mg/kg/d qd) combined with ceftazidime for antiinfection and other adjuvant therapy. Reexamination by Chest computed tomography showed a little inflammation in both lungs and pulmonary interstitial changes significantly reduced (Fig. 1B). A review of cranial magnetic resonance imaging displayed slightly wide sulcus in part of bilateral cerebral hemispheres and disappearance of abnormal signal in the paraventricular white matter area (Fig. 2B).

On the 23rd day after admission, *B pertussis* nucleic acid in the blood was again negative, CSF laboratory biochemistry tests were normal, vital signs were stable, and the infant was discharged on medication (azithromycin 10 mg/kd/d qd for 5 days) and followed up at the outpatient department. Upon outpatient follow-up 2 months after hospital discharge, both neurological and respiratory system examinations of the infant were normal, Regular ECG monitoring did not show prolonged Q-T interval and echocardiography was normal without pulmonary hypertension. Azithromycin was discontinued after a total of 10 days.

## 3. mNGS and PCR methods

The samples were transferred to Hugobiotech (Beijing, China) for mNGS detection. DNA was extracted from the blood and CSF samples using QIAamp DNA Micro Kit (QIAGEN, Hilden, Germany), and the libraries were built using QIAseqTM Ultralow Input Library Kit (Illumina, CA) according to the manual. The quality of libraries was estimated using Qubit (Thermo Fisher, MA) and Agilent 2100 Bioanalyzer (Agilent Technologies, Santa Clara), and qualified libraries were sequenced on Nextseq 550 platform (Illumina, CA). After removing all short, low quality, and low complexity reads and human reads, the remaining reads were finally aligned to National Center for Biotechnology Information (NCBI) Microbial Genome Databases.

For PCR, the insertion sequence of *B pertussis* IS481 gene (145 bp) was amplified using primers: 5’-GATTCAATAGGTTGTATGCATGGT-3’ and 5’-TGGACCATTTCGAGTCGACG-3’. Negative and positive controls were set up for each PCR reaction. PCR products identified as positive by gel electrophoresis were sequenced.

## 4. Discussion

*B pertussis* is a Gram-negative pathogen usually obtained from respiratory specimens. In this case, *B pertussis* was detected in both blood samples and CSF samples, as opposed to the traditional detection from throat swabs, sputum, or alveolar lavage samples. In the blood, pertussis bacteremia can promote the release of a large number of pertussis toxin (PT), an important toxin released by *B pertussis*, resulting in abnormal increase of WBC, which indirectly leads to pulmonary hypertension.^[6,7]^ PT can also penetrate human microvascular cells and promote the migration of macrophages and monocytes across the endodermis of human microvascular, temporarily destroying the integrity of the blood-brain barrier and leading to the occurrence of pertussis encephalopathy.^[8]^ This may provide evidence to elucidate the mechanism of pertussis related complications in our case.

Several risk factors are strongly associated with fatal pertussis in infants, the strongest risk factor for death was extreme leukocytosis, which may cause refractory severe pulmonary hypertension.^[7]^ Retrospective studies also suggested that blind use of hormones and nitric oxide might increase the mortality of severe pertussis.^[7,9]^ In addition, prematurity, age <2 months, and fever (>37.5°C) are associated with the risk of pertussis severity.^[10,11]^ Low birth weight is also an independent risk factor for pertussis in infants.^[12]^ Young age, pulmonary infection, apnea with anoxia are high risk factors for pertussis encephalopathy.^[9,13]^ In this case, the patient is meeting the high risk factors for young age and pulmonary infection.

It has been reported that vaccination of pertussis during pregnancy can effectively reduce incidence of severe pertussis in infants. Many countries have recommended Tetanus-diphtheria-acellular pertussis for women during pregnancy since 2011,^[14–17]^ which can prevent pertussis disease in infants and is safe for both mother and fetus.^[18,19]^ However, routine pertussis vaccination during pregnancy is not yet recommend by the Chinese Center for Disease Control and Prevention at present. In this case report, the mother did not receive pertussis vaccine during pregnancy. The occurrence of pertussis in the infant indicated that on the premise of ensuring the safety of pregnant mothers and fetuses, the incorporation of the maternal pertussis immunization program into Chinese immunization plan should be considered.

Children with pertussis have several significant laboratory abnormalities. The first is a significant increase in WBC and is associated with poor outcomes.^[13]^ In a retrospective study of diagnosed pertussis cases, leukocytosis and lymphocytosis were found in these patients.^[20]^ Among the enrolled 127 children with severe pertussis, patients <3 months of age were found to be more severely ill, and WBC were significantly higher in nonsurvivors.^[21]^ Pertussis encephalopathy, like pulmonary hypertension, is one of the most serious complications of pertussis. The neurological complications of pertussis encephalopathy have various manifestations. Convulsion is the common clinical manifestation of pertussis encephalopathy, and other clinical manifestations include partial paralysis, ataxia, aphasia, blindness, deafness and cerebral rigidity. Kundrat SL et al once put pertussis complicated with encephalopathy into the characteristics of malignant pertussis.^[22]^ Pertussis encephalopathy has a high fatality rate. Mikelova K in Canada showed that symptoms of pertussis encephalopathy were present in 21% of pertussis deaths.^[23]^ And the mechanism of pertussis encephalopathy is complex, the direct effect of pertussis infection toxin is related to pertussis encephalopathy, hypoxia caused by a variety of reasons can also aggravate the progression of pertussis encephalopathy. At present, there is still a lack of effective studies to confirm that pertussis encephalopathy can eventually lead to specific changes in cranial imaging. Because the mechanism of pertussis encephalopathy is complex and the neurological symptoms and imaging are not specific, the empirical identification of pertussis encephalopathy is often based on its symptoms and high-risk factors. However, on the basis of, meeting the clinical manifestations of pertussis encephalopathy, this case successfully found *B pertussis* in CSF by mNGS for the first time, which made the diagnostic evidence of pertussis encephalopathy more sufficient.

The diagnosis can be made by culture, and the pathogen can also be detected by specific antibody or specific PCR assay. Among laboratory detection methods for *B pertussis*, culture and PCR methods are suitable for screening pertussis that occurs within 2 weeks, and PCR is more sensitive than culture.^[24]^ However, both culture and PCR are prone to false negative results when the course is more than 2 weeks. Compared with culture and PCR methods, mNGS allows for early and accurate screening for extrapulmonary pertussis, and still has high sensitivity in the middle and late stages of infection. Moreover, mNGS can objectively, comprehensively and unbiased detect all pathogenic microorganisms in clinical samples, including viruses, bacteria, fungi, and parasites.^[25]^ Considering the low sensitivity of conventional methods using CSF, mNGS has great advantages to clarify the presence of *B pertussis* in the Central Nervous System, providing more evidences to adjust the treatment.

This patient was initially diagnosed with severe pneumonia, intracranial infection, and sepsis, and was first treated with meropenem combined with vancomycin, but his condition continued to deteriorate. After an initial empirical high level of combination anti-infective therapy (antibacterial and antifungal) to prevent worsening of the infection and even septic shock, the patient was transitioned to targeted therapy to eradicate the pathogen. Treatment was rigorous.

To our knowledge, this is the first case report of infant pertussis encephalopathy diagnosed by mNGS, emphasizing the importance of mNGS using CSF for early diagnosis of pertussis-associated encephalopathy, especially for patients with complex clinical symptoms and ineffective empirical treatment.

## Acknowledgments

The authors thank all the clinical staff contributed to the study.

## Author contributions

**Conceptualization:** Zhongqiang Liu.

**Data curation:** Xiao Wang, Han Xia.

**Formal analysis:** Han Xia.

**Funding acquisition:** Zhongqiang Liu.

**Writing – original draft:** Haiyang Zhang.

**Writing – review & editing:** Han Xia.
